# Ancient landscapes of the Namib Desert harbor high levels of genetic variability and deeply divergent lineages for Collembola

**DOI:** 10.1002/ece3.5103

**Published:** 2019-04-10

**Authors:** Gemma E. Collins, Ian D. Hogg, Janine R. Baxter, Gillian Maggs‐Kölling, Don A. Cowan

**Affiliations:** ^1^ School of Science University of Waikato Hamilton New Zealand; ^2^ Polar Knowledge Canada Canadian High Arctic Research Station Cambridge Bay, Nunavut Canada; ^3^ Centre for Microbial Ecology and Genomics University of Pretoria Pretoria South Africa; ^4^ Gobabeb Research and Training Centre Walvis Bay Namibia

**Keywords:** biogeography, Collembola, genetic diversity, Namib Desert

## Abstract

**Aim:**

To assess spatial patterns of genetic and species‐level diversity for Namib Desert Collembola using mitochondrial DNA cytochrome *c *oxidase subunit I (COI) gene sequences.

**Location:**

Namib Desert gravel plains.

**Taxon:**

Collembola (springtails).

**Methods:**

A total of 77 soil samples were collected along NE‐SW (60 km) and E‐W (160 km) transects from within a 4,000 km^2^ area of the Namib Desert gravel plains. We extracted 434 springtails from the 37 samples which contained Collembola and sequenced them at the COI gene locus. In the absence of specific taxonomic keys and previous genetic data for these taxa, we used Generalized Mixed Yule Coalescent (GMYC) analyses to provide putative species‐level designations.

**Results:**

We obtained 341 successful COI sequences, 175 of which were unique haplotypes. GMYC analyses identified 30 putative species, with up to 28% sequence divergence (uncorrected p‐distance). The distribution of genetic variants was disjunct, with 97% of haplotypes and 70% of “GMYC species” found only at single sites.

**Main conclusions:**

Dispersal events, although rare, may be facilitated by environmental events such as prevailing onshore winds or occasional flow of rainwater to the coast. We conclude that the high genetic diversity we observed is the result of ancient springtail lineages, patchy distribution of suitable habitats, and limited dispersal (gene flow) among habitable locations.

## 1 INTRODUCTION

**Figure 1:**
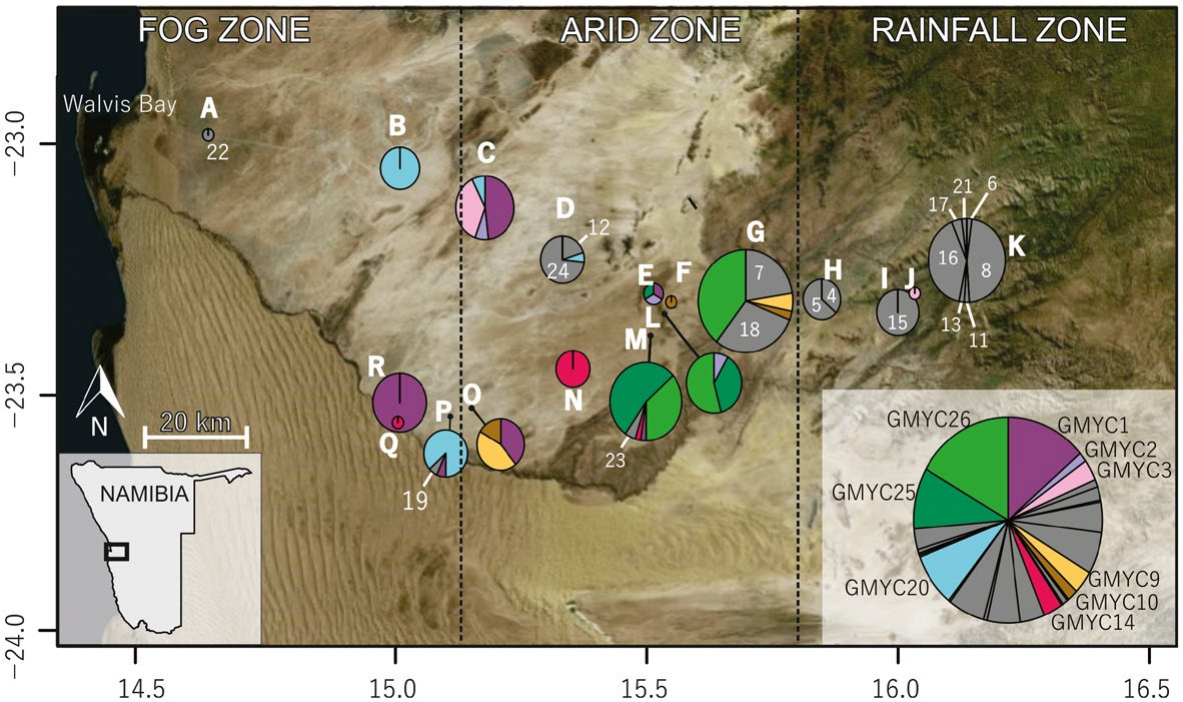
Map showing Namib Desert collection sites for our 336 Isotomid COI sequences (582 bp) grouped into the 26 GMYC species and coloured accordingly (excluding Bourletiellidae, Sminthuridae and Neanuridae haplotypes). This figure was created in RStudio using the packages OpenStreetMap (Fellows & Stotz, 2016) and mapplots (Gerritsen, 2014) and built‐upon in CorelDraw X5. Grey colouring indicates GMYC species that were found at only one site. Pie charts are centred at collection sites (or designated by black markers where they overlapped) and sizes of pie charts are proportional to the total number of individuals sequenced from each site. Inset legend indicates colour of each GMYC species that was found at more than one site, and also shows overall proportions of each GMYC species. Colours correspond to Figure 2 which provides additional phylogenetic information. Sites A‐K include those in the west‐east transect (following the C14 road), whereas Sites L‐R follow a northeast‐southwest transect. Generalised aridity zones (fog, arid and rainfall) are shown in relation to sampling sites (Scola et al. 2018)

The capacity to support eukaryotic life is limited within both hot and cold deserts owing to extreme temperatures, high ultraviolet radiation, high winds, and low nutrient and water availability (Ayal, 2007; Polis, 1991). Consequently, the number of species, community structure, and ecosystem functions are often restricted and driven primarily by abiotic factors (Hogg et al., 2006; Holm & Edney, 1973; Sømme, 1995). The Namib Desert is long (2,000 km) and narrow (50–150 km), bounded by the Atlantic Ocean on the west and the Great Escarpment of southern Africa on the east (Henschel & Lancaster, 2013). Gravel plains of the Central Namib occur between the Kuiseb (south) and Ugab (north) ephemeral rivers. Limited moisture in the gravel plains is provided through marine fog events in the west and sporadic rain at higher elevations in the east, leaving a hyperarid area between 60 and 90 km from the coast (Eckardt et al., 2013). As such, the gravel plains can be categorized into three xeric zones (fog, arid, and rainfall; Figure 1) occurring along a gradient from the coast to the Escarpment (~1,000 m asl) (Scola et al., 2018).

In addition to aridity, other physical and chemical parameters within the gravel plains also vary with distance from the coast, generally corresponding with the xeric zones. Scola et al. (2018) found significant differences (ANOVA, *p* < 0.05) between zones for 13 of 17 measured physical and chemical parameters, based on sampling of a 190 km longitudinal transect at 10‐km intervals (*n* = 20 sites). For example, soils in the coastal fog zone contained higher levels of sodium, calcium, and potassium than soils in the arid and rainfall zones, whereas soils in the rainfall zone contained more organic matter and phosphorus. Bacterial diversity was also significantly different between zones (PERMANOVA, *p* < 0.05; T‐RFLP analyses of 16S rRNA community fingerprints), and enzyme activity increased for sites further inland, suggesting that gradients in the cycling of carbon, nitrogen, and phosphorus may also exist. However, water was still identified as the most important driver of bacterial diversity and function (Scola et al., 2018), although in an Austrian dry grassland it was vegetation, soil temperature, and soil texture that were the strongest drivers of springtail community composition (Querner et al., 2018).

While relationships between environmental gradients and microbial (e.g., Johnson, Ramond, Gunnigle, Seely, & Cowan, 2017; Scola et al., 2018; Stomeo et al., 2013) and plant (Hachfeld, 2000) communities within the Namib Desert are partly understood, only limited data are available for the invertebrate fauna and most of this research has focused on the macroarthropod taxa. In a study of ant species across the Namib Desert, a strong correlation (*r* ^2^ = 0.94) was found between mean annual rainfall (and therefore productivity) and number of ant species, with most ant species present in the eastern (and more productive) end of the transect (Marsh, 1986). Crawford and Seely (1994) found that recent rainfall at a site near Homeb was associated with greater arthropod abundance and species richness in pitfall trap collections, as compared to other Namib dune sites, although no collembolans were reported in this study. Furthermore, in a study of the decomposition of litter bags buried in the Namib Desert dunefield, no microarthropods were reported (Crawford & Seely, 1994).

Previous studies of microarthropods have been focused on collembolan diversity and led to the identification of four species; *Willemia namibiae* (Thibaud & Massoud, 1988), *Folsomides angularis* (previously named *Isotoma angularis*; Axelson, 1905), *Folsomides parvulus* (Stach, 1922), and *Friesea* sp. Specimens of *Folsomides cf. angularis* were obtained from soil samples in the gravel plains, specifically from soil associated with *Euphorbia*, *Stipagrostis ciliata,* and *Welwitschia mirabilis* plants (André, Noti, & Jacobson, 1997). In a study of the arthropods associated with *Welwitschia*, Collembola were found to dominate the soil fauna at only one site (Marsh, 1987). However, little other work on the Namib Desert microarthropods has been undertaken.

While complex biological communities can persist in pockets of suitable microhabitat within extreme landscapes, dispersal processes tend to support genetic homogenization. However, dispersal is a considerable challenge for microarthropods, particularly in desert ecosystems (Cracraft, 1994; Stevens & Hogg, 2002). Possible dispersal mechanisms include wind, water, human intervention, zoochory (Greenslade & Convey, 2012; Gressitt, Leech, Leech, Sedlacek, & Wise, 1961), and ground‐based movement (walking or jumping; (Åström & Bengtsson, 2011). Desolate areas surrounding “island” habitats can act as barriers to dispersal and restrict gene flow. Isolation leads to the accumulation of genetic mutations within local populations, subsequently exhibited as distinct genetic patterns across the landscape. Accumulation of allopatric mutations in local populations occupying microhabitats, such as organic‐rich soils around plants or under quartz hypoliths, is likely to be high (Stomeo et al., 2013; Wong et al., 2010).

Here, we examined the diversity of Collembola within the Namib Desert using an analysis of cytochrome *c* oxidase subunit I (COI) sequences. Sequencing of the COI locus has been demonstrated to be an effective method for resolving population‐level and species‐level differences for Collembola, as well as identifying cryptic species (e.g., Hogg & Hebert, 2004; Porco, Bedos, & Deharveng, 2010; Raschmanová et al., 2016). Previous studies using multiple markers, including COI, have shown that COI is likely to be representative of overall genomic differences and is an appropriate measure of genetic differentiation between populations (e.g., Hoppeler et al., 2016; Stevens & Hogg, 2003; Saltzwedel, Scheu, & Schaefer, 2016). Here, we determine genetic variability, using COI, for Collembola across large‐scale Namib Desert soil transects, and assess the spatial distribution of COI haplotypes and putative species‐level groupings as an indication of population connectivity and dispersal.

## 2 MATERIALS AND METHODS

### 2.1 Sample collection

Samples were collected from within the gravel plains of the Namib Desert using a previously established 190 km sampling transect following the C14 national road from west to east (Marsh, 1986; Scola et al., 2018) at roughly 20‐km intervals (Sites A‐K), and from sites along a 60‐km NE‐SW transect (Sites L‐R; Figure 1, Supporting information Appendix S1). At each site, 1–5 soil samples (approximately 500 g each) were taken to a depth of 10 cm using an alcohol‐wiped hand trowel from under quartz rocks (3–30 cm dia.) with obvious hypolithic algal growth (observed as a green ring of growth around the lower margin and undersurface of the rock) and beneath vegetation where present. At each site, all samples were taken from within approximately a 20‐m radius. When both quartz and plants were present at a particular site, we took separate samples from each habitat and pooled the sequences into their respective sites (A ‐ R), for the purposes of the analyses. Eighteen samples were taken from 12 April to 17 April 2015, and an additional 59 samples were taken from 11 April to 15 April 2016 to include a wider range of sites and habitat types. Soil samples were returned to the Gobabeb Research and Training Centre (GRTC) for the extraction of specimens via flotation in tap water (after Edwards, 1991; Macfadyen, 1953). Individual Collembola specimens were removed with the aid of dissecting stereomicroscopes (Olympus SZ40 and Stemi 2000 ZEISS) and immediately preserved in 100% ethanol for subsequent analyses.

### 2.2 Genetic analyses

Images for each individual specimen were taken at 40× magnification using either a Nikon Coolpix 4500 or AxioERc5s ZEISS camera attached to the stereomicroscopes for morphological reference. Individuals were then loaded into single wells on 96‐well microplates for processing at the Canadian Centre for DNA Barcoding (CCDB). A further 24 specimens collected in 2016 were returned to New Zealand for higher‐resolution photography and DNA extraction, PCR amplification, and sequencing at the University of Waikato (UoW). Morphological vouchers (recovery of skins following the DNA extraction process) were also retained for selected morphotypes (specimens NAMIB‐096 to NAMIB‐119).

Total DNA was extracted from specimens following a glass fiber plate method (AcroPrep) (Ivanova, Dewaard, & Hebert, 2006) at CCDB or using a REDExtract‐N‐Amp kit (Sigma‐Aldrich) at UoW. A 658‐bp region of the COI gene was targeted for amplification using the universal primers LepF1 (5′‐ATTCAACCAATCATAAAGATATTGG‐3′) and LepR1 (5′‐TAAACTTCTGGATGTCCAAAAAATCA‐3′) (Hajibabaei, Janzen, Burns, Hallwachs, & Hebert, 2006) at UoW, and these primers were used in combination with HCO2198 (5′‐TAAACTTCAGGGTGACCAAAAAATCA‐3′) and LCO1490 (5′‐GGTCAACAAATCATAAAGATATTGG‐3′) (Folmer, Hoeh, Lutz, & Vrijenhoek, 1994) at CCDB. Thermocycling at CCDB involved an initial denaturation at 94°C for 1 min followed by five cycles (94°C for 1 min, 45°C for 1.5 min, and 72°C for 1.5 min) then 35 cycles (94°C for 1 min, 50°C for 30 s, and 72°C for 1 min) of denaturation and polymerase amplification, with a final elongation at 72°C for 5 min. At UoW, thermocycling conditions were altered to an annealing temperature of 51°C for 1.5 min during each of the 35 cycles. Successful amplification products were cleaned with Sephadex (Sigma‐Aldrich) at CCDB and Exonuclease I and shrimp alkaline phosphate (illustra ExoProStar 1‐Step) at UoW. Sequencing was carried out in forward and reverse directions on an ABI 3730xl DNA Analyzer (Applied Biosystems) at CCDB and an ABI 3130 at UoW. Edited sequences along with raw trace files, specimen images, and collection details were all uploaded to the Barcode of Life Datasystems (BOLD) database (Ratnasingham & Hebert, 2007) under the dataset “DS‐NBCOL” and cross‐referenced to GenBank under accession numbers MK402325 to MK402665. Sequences were assigned a Barcode Index Number (BIN) using the algorithm on BOLD (Ratnasingham & Hebert, 2013).

### 2.3 Data analyses

A total of 434 individuals were analyzed, and 341 useable sequences were obtained. Of these, 294 (86%) covered the complete 658 bp barcoding gene region while the remaining 47 sequences varied from 618 to 642 bp in length. An alignment of only full‐length (*n* = 294; 658 bp) reads was compared with a shorter alignment containing all sequences (*n* = 341; 582 bp) for assessment of haplotype diversity and coverage (data not shown). We prioritized additional sequence coverage provided by the shorter alignment over the loss of individual data which would occur by maximizing alignment length. As such, the final alignment was trimmed to 582 bp for inclusion of all 341 springtail sequences from April field seasons of 2015 and 2016. All alignments were generated using MUSCLE 3.8.425 (Edgar, 2004) as implemented in Geneious v7.1.9 (Kearse et al., 2012; http://www.geneious.com).

A search for available sequences was made on BOLD and GenBank for the four springtail taxa previously reported from the Namib Desert; *Willemia namibiae*, *Folsomides angularis*, *Folsomides parvulus,* and *Friesea* sp. No sequences were found for *W. namibiae *or *Friesea* specific to the Namib Desert. However, one sequence was available for *F. parvulus *(China) and we were able to gain access to the two sequences on BOLD for *F. angularis *(unpubl. data), from Moldova (L. Deharveng and G. Buşmachiu; COLLH3578‐11) and Norway (A. Fjellberg; NORCO462‐15). As such, all sequences available for the genus *Willemia* (KU373861, KU373867, KF642103, HQ732083), along with selected *Friesea* sequences (NC_010535, EU124719, KF642199) and the *F. parvulus *(JN981069) and *F. angularis *(NORCO462‐15, COLLH3578‐11) sequences were downloaded to Geneious and included in the alignment to add context for phylogenetic tree constructions.

The final 582 bp alignment was assessed in jModelTest v2.1.4 (Darriba, Taboada, Doallo, & Posada, 2012; Guindon & Gascuel, 2003), and a Maximum Likelihood (ML) phylogenetic tree was generated in MEGA v7 (Kumar, Stecher, & Tamura, 2016) with GTR+G+I and 1000 bootstrap samples. A pairwise distance matrix was also generated in MEGA to give divergence values between unique sequences, as well as within and between sites, based on uncorrected p‐distances (Collins & Cruickshank, 2013 and references therein). Chi‐square tests as implemented in PAUP* 4.0b (Swofford, 2003) were used to determine whether base frequencies were equal among all sites, parsimony‐informative sites and third codon positions for all sequences. PAUP* was also used to determine the number of variable and parsimony‐informative sites in the alignment (excluding outgroups).

In the absence of detailed taxonomic evaluation, Generalized Mixed Yule Coalescent (GMYC) analyses (Monaghan et al., 2009; Pons et al., 2006) were used alongside BIN designations to estimate species‐level diversity. While both methods have been shown appropriate for grouping sequences to species level (Hausmann, Haszprunar, & Hebert, 2011; Leliaert, Verbruggen, Wysor, & Clerck, 2009), we found extremely high genetic variability using the BIN algorithm (*n* = 43) relative to the GMYC analyses (*n* = 30). As BIN designations were not always monophyletic (e.g., ACW5608), we focused primarily on the GMYC method for grouping sequences into genetic lineages (i.e., resembling “species‐level” diversity) for subsequent analyses which has similarly been done for the European springtail *Parisotoma natabilis *(Saltzwedel, Scheu, & Schaefer, 2017). For assessing fine‐scale haplotype‐level variability, we assigned a code (e.g., NC‐001) to each unique sequence obtained (*n* = 175).

For GMYC analyses, we used a Bayesian framework for estimating the tree which was then assessed for potential species‐level designations through GMYC analyses in R v3.5.1. To create the Bayesian tree, our final alignment (341 sequences, 582 bp) was exported from MEGA in nexus format, priors were chosen in BEAUTi v2.4.7 (100 million MCMC chain length, strict clock, yule model, GTR+G+I), the analysis was run in BEAST v2.4.7 and the resulting “.trees” file and posterior distribution were summarized in TreeAnnotator v2.4.7 (10% burn‐in), after checking the log file in Tracer v2.4.7 for effective sample size (posterior ESS of 4,396, likelihood ESS of 2,420) (Bouckaert et al., 2014). The summarized tree was then imported to R for GMYC analysis which utilized the packages “ape” (Paradis, Claude, & Strimmer, 2004), “paran” (Dinno, 2012), “rncl” (Michonneau, Bolker, Holder, Lewis, & O'Meara, 2016), and “splits” (Ezard & Fujisawa, Tomochika Barraclough, 2017).

We also used R for generating haplotype pie charts, centered at their respective collection coordinates. For these analyses, haplotypes NC‐021, NC‐150, NC‐097, NC‐098, and NC‐139 were excluded due to their “outgroup” position in the ML tree (i.e., Bourletiellidae, Sminthuridae, and Neanuridae). Haplotypes were grouped according to “species‐level” GMYC designations (*n* = 26), and the packages “OpenStreetMap” (Fellows & Stotz, 2016) and “mapplots” (Gerritsen, 2014) were used to visualize the distribution of these putative species across sample sites.

## 3 RESULTS

**Figure 2:**
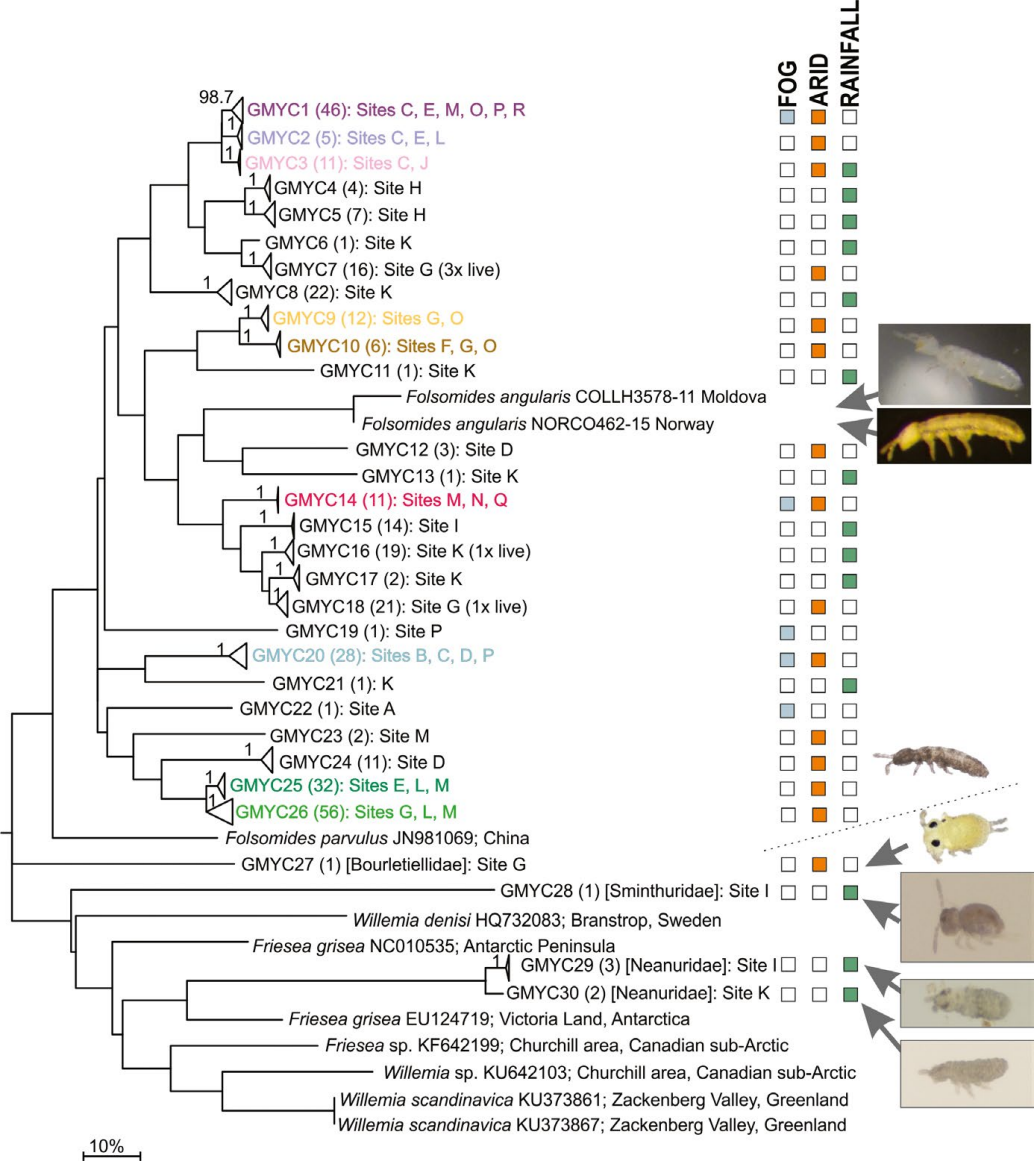
Maximum Likelihood tree (MEGA; GTR+I+G) based on the trimmed (582 bp) alignment of Namib Desert Collembola, including our 175 unique COI sequences as well as selected sequences from GenBank. Clades have been collapsed to ‘GMYC species’‐level and supporting bootstrap values (1000 replicates), where >50%, have been provided for clades. Number of individuals within each collapsed clade are shown in parentheses. Coloured squares indicate which of the three aridity zones across the Namib Desert that each GMYC species was found, and representative images are provided for the main morphogroup (i.e. Isotomidae) as well as for each of the outgroup taxa obtained in the current study. See Supporting information Appendix S4 for expanded version of this tree

We found springtails in 37 of the 77 soil samples and 434 individuals were recovered, from which 341 COI sequences were successfully obtained (79% sequencing success). At least ten individuals were noted to be alive (moving) at the time of sample processing, and six of those individuals, which were from sites G and K, were sequenced successfully (Table 1; Figure 2). Other specimens that were not observed moving were possibly either dead or in an anhydrobiotic (dried) metabolic state. Supporting information Appendix S3 provides a list of samples that did not contain springtails and also samples where springtails were extracted but failed to yield sequence. The final trimmed alignment covered a 582 bp region of the *Drosophila melanogaster* mitochondrial genome (GenBank Accession Numbers: X03240, NC_001322) including nucleotides 1551 (3rd codon position) to 2132 (2nd codon position). No stop codons, insertions, or deletions were detected, indicating that sequences were of mitochondrial origin and not nuclear pseudogenes. The alignment contained 269 variable sites (250 parsimony‐informative), and 175 unique haplotype sequences were identified.

**Table 1:** Coordinates for each site within the Gravel Plains of the Namib Desert from which springtail COI sequences were obtained, including number of sequences (*n*) and those of which were obtained from live specimens in parentheses, unique haplotypes (*h*), and number of putative species (as determined by GMYC analyses)

Site	*n *(live)	*h*	*n* GMYC species	Latitude	Longitude	Elevation (m)
A	1	1	1	−23.00152	14.66968	121
B	12	4	1	−23.06637	15.0421	539
C	27	14	4	−23.14309	15.20738	623
D	15	9	3	−23.24377	15.35777	757
E	3	3	3	−23.31041	15.53481	946
F	1	2	1	−23.326	15.569	959
G*	72 (4)	58	6	−23.32406	15.71384	897
H	11	6	2	−23.32093	15.86175	969
I*	18	6	3	−23.34627	16.00951	1,097
J	1	1	1	−23.30919	16.04211	1,053
K*	49 (2)	23	8	−23.24533	16.14359	1,268
L	24	18	3	−23.34888	15.55568	978
M	41	30	5	−23.391	15.529	884
N	9	1	1	−23.456	15.378	774
O	18	7	3	−23.53272	15.18375	556
P	15	7	3	−23.54804	15.14017	518
Q	1	1	1	−23.56	15.038	406
R	23	4	1	−23.52144	15.04181	436
Total	341	(175)	(30)			

Note

Sites where nonisotomid taxa were found are denoted with asterisks.

There was an overall A‐T bias of 62.8%. However, chi‐square tests confirmed homogeneity of base frequencies across all sites as well as constant, variable, and informative sites, and at first and second codon positions (*p* > 0.1). Base frequencies were not homogeneous at the third codon position (A‐T = 79%; X^2^ = 1,345, *df* = 963, *p* < 0.05), supporting the use of general time reversible plus gamma distribution and proportion of invariable sites (GTR+G+I; e.g., Tavaré, 1986) in ML tree construction as identified by JModelTest as the most likely model of sequence evolution, given the sequence data (‐lnL = 8896.99).

The two available COI sequences of *Folsomides angularis *(COLLH3578‐11 and NORCO462‐15) were highly divergent (>17%) from any of the Namib Desert sequences presented here (Figure 2). However, they grouped out in the center of the main dataset (Figure 2) which suggests the Namib Desert specimens are more closely related to *Folsomides angularis *(type locality Finland) than any other species with sequence data available online. While *Folsomides parvulus* has been previously recorded from the Namib Desert (Thibaud & Massoud, 1988), the two COI sequences on GenBank for this species (384 bp; 658 bp) did not closely match any of our sequences (GenBank search < 85% similarity). Similarly, the four available COI sequences for the genus *Willemia*, as well as three representative *Friesea* sequences, also did not closely match any of the Namib Desert springtail sequences (<85% similarity; Figure 2).

In addition to the main dataset (Isotomidae), five sequences identified as belonging to the families Bourletiellidae (NC‐021), Sminthuridae (NC‐150), and Neanuridae (NC‐097, NC‐098, NC‐139) and were distinct (maximum divergences 27%–28.2%) in the ML tree (Figure 2). Excluding these nonisotomid species, maximum divergence (uncorrected p‐distance) between any two sequences was 22% (NC‐047 vs. NC‐028/037). Minimum divergence between our sequences and GenBank or BOLD sequences included in the alignment (*n* = 10) was 17% (NC‐141 and *F. angularis*).

### 3.1 Haplotype‐level diversity

The most abundant haplotypes observed were NC‐074 (*n* = 15), NC‐093 (*n* = 15), and NC‐095 (*n* = 11) which were each found at single sites only (Sites R, K, and I, respectively). Most of the remaining haplotypes were singletons (118 of 175) (see Supporting information Appendix S2 for further detail). Only six haplotypes were found at more than one site, each of which was found at two sites only. Haplotype NC‐040 was the most distantly spaced, occurring at Sites C and J which were 87 km apart, whereas the other two shared haplotypes NC‐032 and NC‐042 were each found at locations that were only 5 km apart (Sites O‐P and L‐M, respectively). Although the sharing of identical sequences between locations was extremely rare, haplotype sharing was found in only east–west (NC‐006(2), NC‐040(8)) or northeast‐southwest (NC‐001(10), NC‐015(7), NC‐032(2), NC‐042(3)) directions. No haplotypes were shared directly in the north–south direction.

### 3.2 Species‐level diversity

Generalized mixed yule coalescent analyses indicated 26 putative species within our dataset of 334 isotomid sequences, with an additional four species identified from among the outgroup taxa (*n* = 7) sequenced (Bourtiellidae, Sminthuridae, Neanuridae). Sequence coverage ranged from one to 56 sequences per GMYC species and mean pairwise p‐distance between GMYC species ranged from 4.3% to 21.8% (intraspecific p‐distance max. 3.6%). Of the 26 GMYC species identified, 23 were in agreement with BIN designations and the remaining three species (GMYC5, 8, and 26) contained more than one BIN each (Table 2; see Appendix S2). We grouped our sequences according to these GMYC designations for species‐level comparisons.

**Table 2:** Details for each putative species of springtail (Collembola), as identified by GMYC analyses, including number of sequences (*n*), number of haplotypes (*h*), mean intraspecific divergence (uncorrected p‐distance), and sites where each species was found

GMYC species	BINs	*n*	*h*	Mean intraspecific divergence	Sites	Direction of sharing
GMYC1	ADB5701	46	15	1.38%	C(13),E(1),M(1),O(7),P(1),R(23)	North–south and east–west
GMYC2	ADB6383	5	4	1.00%	C(2),E(1),L(2)	East–west
GMYC3	ADB6305	11	3	0.23%	C(10),J(1)	East–west
GMYC4	ADB6302	4	3	1.03%	H	‐
GMYC5	ADB6299, 6555, 6301	7	3	2.63%	H	‐
GMYC6	ADC0536	1	1	‐	K	‐
GMYC7	ACU5054	16	13	0.82%	G	‐
GMYC8	ADB6300, 6378	22	2	3.61%	K	‐
GMYC9	ACU5273	12	5	0.82%	G(4),O(8)	Northeast–southwest
GMYC10	ACU4213	6	3	0.69%	*F*(1),G(2),O(3)	Northeast–southwest
GMYC11	ADC0533	1	1	‐	K	‐
GMYC12	ADB6633	3	1	‐	D	‐
GMYC13	ADC0535	1	1	‐	K	‐
GMYC14	ACU5084	11	2	0.17%	M(1),N(9),Q(1)	Northeast–southwest
GMYC15	ADB5714	14	3	0.23%	I	‐
GMYC16	ADB6379	19	10	1.39%	K	‐
GMYC17	ADC0534	2	2	1.55%	K	‐
GMYC18	ADB6047	21	14	1.29%	G	‐
GMYC19	ADB6381	1	1	‐	P	‐
GMYC20	ADB6632	28	10	1.65%	B(12),C(2),D(1),P(13)	North–south and east–west
GMYC21	ADB6303	1	1	‐	K	‐
GMYC22	ADB6384	1	1	‐	A	‐
GMYC23	ACU4253	2	1	‐	M	‐
GMYC24	ADB6382	11	6	1.53%	D	‐
GMYC25	ACU4405	32	23	1.01%	E,L,M	‐ (<10‐km radius)
GMYC26	ACU4502, 164‐166; ACW5240, 5553, 5554, 5606–8; ADB9904	56	41	2.70%	G(28),L(13),M(15)	‐ (<10‐km radius)
GMYC27	ACW5615	1	1	‐	G	‐
GMYC28	ADB6773	1	1	‐	I	‐
GMYC29	ADC0483	3	2	‐	I	‐
GMYC30	ADB6048	2	1	‐	K	‐

Note

Barcode Identification Numbers (BINs) were congruent with GMYC designations in most cases, excepting GMYC5, 8, and 26 where the GMYC analysis grouped multiple BINs as one putative species. Notes on spatial arrangement of sites (direction of sharing) are also provided. See Supporting information Appendix S2 for further haplotype details.

Each GMYC species was found at between one and six sites. However, most species (17 of 26) were found at only one site each (Table 2; Figure 2). The most distantly spaced was GMYC3 which was found at both Sites C and J (87 km), and the species found at the most sites was GMYC1 (Sites C, E, M, O, and P). The highest species diversity was found at Site K, which contained seven species; none of which was found at any other site. Even though GMYC25 and GMYC26 were found in high abundance (*n* = 32 and *n* = 56, respectively), they were each only found at sites within approximately a 10‐km radius (E‐L‐M and G‐L‐M, respectively).

The proportion of GMYC species that were endemic to each of the three xeric zones was highest for the rainfall zone (90.9%), intermediary for the arid zone (71.4%), and lowest for the fog zone (40%). However, the total number of sequences (range 52–210) and GMYC species (range 5–14) obtained within each zone is highly variable (Table 3). See Supporting information Appendix S2 for further detail.

**Table 3:** Percentage of GMYC species endemic to each of the three xeric zones across the Namib Desert

Xeric zone	Sites	Total *n *GMYC species in each zone (*n* sequences)	Endemic GMYC species	*n* endemic GMYC species (*n *sequences)	Percentage endemic GMYC species (%)
Fog	A, B, P, Q, R	5 (52)	19, 22	2 (2)	40
Arid	C–G, L–O	14 (210)	2, 7, 9, 10, 12, 18, 23 – 26	10 (164)	71.4
Rainfall	H–K	11 (79)	4, 5, 6, 8, 11, 13, 15, 16, 17, 21	10 (72)	90.9

## 4 DISCUSSION

Based on our COI dataset for Namib Desert springtails, we found evidence for highly restricted distribution of distinct genetic lineages (“GMYC species”) and limited evidence for present‐day dispersal (and gene flow) among sites. From 334 isotomid sequences, we detected the potential for up to 26 distinct species (GMYC analyses; >4.3% p‐distance), 17 of which were found at only single sites. Furthermore, only six of the 170 haplotypes were found at more than one site and never at more than two sites. Due to this high genetic diversity and lack of apparent dispersal between sites, we suggest that the extreme Namib Desert environment supports a unique and highly diverse assemblage of springtails.

A lack of global understanding around soil animal taxonomy was highlighted by Rougerie et al. (2009), where only 44 species of Collembola had been morphologically identified from a dataset of 695 sequences. Of the identified specimens, high levels of cryptic diversity (15% divergence) were found. Similarly, for the Namib Desert, *Folsomides angularis *was the closest available morphological identification (>17% divergence) for the 26 potential cryptic species of Collembola from within the dataset of 341 COI sequences presented in the current study. We did not morphologically identify our sequenced individuals as no specific taxonomic references were available for Namib Desert Collembola. However, a complementary morphological study (Baxter, 2018), using European taxonomic keys, identified individuals from the genus *Folsomides*, family Entomobryidae, and order Symphypleona (J. Baxter and C. Janion‐Scheepers unpubl. data).

The two available sequences on BOLD for *F. angularis *from Norway and Moldova were grouped within our sequences (>17% divergent). Collembola found in previous Namib Desert surveys (André et al., 1997; Thibaud & Massoud, 1988) were identified as *Folsomides cf. angularis*. However, many species of *Folsomides* have been described from other desert environments (e.g., Suhardjono & Greenslade, 1994). It is unlikely that those found in the Namib Desert are the same as the European *F. angularis* (type locality Finland), but rather they are morphologically similar variants that have adapted in situ. In addition, high genetic divergences within our *Folsomides* specimens (<22%) are suggestive of a possible species complex or cryptic speciation (in agreement with suggestions made in other Namib Desert studies; e.g., André et al., 1997; Thibaud & Massoud, 1988). Such marked genetic differences are perhaps not surprising when considering the environmental gradients across which we sampled (i.e., soil properties, nutrient and water availability, and aridity).

Levels of genetic variability were higher for our Namib Desert *F. cf. angularis* dataset (*n* = 334 sequences; 4.3%–21.8% variability among the 26 GMYC lineages) than for the European springtail *Parisotoma notabilis *which had five COI lineages (15%–18% variability), also determined by GMYC analyses (Porco et al., 2012; Saltzwedel et al., 2017). Similarly, three other European springtail species *Ceratophysella denticulate*, *Folsomia quadrioculata,* and *Isotomiella minor* had high genetic variability between sites (11%–20%) (Saltzwedel et al., 2016). Porco et al. (2012) and Saltzwedel et al. (2017) both used 2.3% divergence per million years for molecular clock estimates, and all four European species had levels of divergence (11%–20%) aligned with Miocene (8.7–5.3 MYA) or late Pliocene (5.3–4.7 MYA) divergence. Furthermore, patterns of population structure in the Namib Desert are similar to these European species, where variability within each of the isolated populations is low (dominated by the founder lineage) while variability between sites is high (suggesting Miocene origin and subsequent differentiation in situ).

As different GMYC species showed differing distributional patterns, we speculate that these groups may exhibit physiological differences or that the drivers of dispersal may vary regionally (e.g., prevailing wind direction or catchment flow profiles). For example, GMYC23‐26 (*n* = 101) are clustered on the phylogenetic tree and also on the map (within 18‐km radius) whereas GMYC1‐3 (*n* = 62) are more widespread (within 50‐km radius). Possible explanations for longitudinal dispersal include onshore winds supporting aerial dispersal from the coast toward the inland mountains (southwest to northeast), where it is likely that individuals in an anhydric (dried) state are able to disperse in wind currents more easily than live individuals. Ephemeral drainage networks facilitate aquatic dispersal from areas of higher elevation toward the coast (east to west), and there is also the possibility of underground or deep soil dispersal (Griebler et al., 2014).

We found that inland sites, particularly Sites H, I, and K, each harbored distinct GMYC species with no sharing between sites, whereas in the fog and arid zones species were more widespread. In most cases where GMYC species were found at more than one site, a greater number of individuals were found at the more western sites within their respective distributional ranges. Based on these findings, we suggest the inland locations may be providing source localities for collembolan diversity, with individuals possibly dispersing toward the coast (Freeman, 2009; Glick, 1939; Johnson, 1957).

From within our dataset of 341 COI sequences, we found a high proportion of single haplotypes, with 118 of 175 haplotypes (67%) represented by single sequences. Furthermore, we obtained more sequences from Site G (*n* = 72; 21% of all sequences) than from any other site and this directly resulted in Site G also having the highest haplotype diversity (58 haplotypes). Further sampling may therefore reveal additional diversity and haplotype sharing among locations. Non‐isotomid taxa (GMYC27‐30) were all found at inland sites only (G, I, and K), and we suggest that the generally wetter and more organically rich inland sites may provide more favorable conditions to support a wider range of taxa than more coastal sites where only isotomids were found. In support of this finding, a study of the distribution of ant species across a similar Namib Desert transect identified greater species richness at inland sites, where mean annual rainfall and productivity was higher than at more coastal sites (Marsh, 1986). Furthermore, coastal soils have elevated levels of cations (Scola et al., 2018), indicative of high salinity, and many of our coastal samples did not contain springtails (see Supporting information Appendix S3). The occurrence of dew within soils would also influence the availability of springtail habitat (Belnap & Lange, 2003).

While inland sites (H‐K) receive more rainfall days per year as compared to the arid (Sites C‐G, L‐O) and fog (Sites B, P‐R) zones (Scola et al., 2018), we found no evidence to support present‐day dispersal of springtails between inland sites. This is surprising, as greater water availability in general may have increased springtail dispersal capabilities and likelihood of survival in‐transit. However, based on these findings, we highlight the importance of microhabitat environments, such as the hypolithic algal, bacterial, and fungal communities found under quartz rocks (e.g., Makhalanyane et al., 2013), for supporting springtail diversity. With climate changes predicted to exacerbate desertification, isolation may become even more pronounced and genetic mutations may continue to accumulate in these “island” populations (Lioubimtseva, 2004; Makhalanyane et al., 2015), with Namib Desert springtails possibly facing even greater challenges to their long‐term persistence.

## 5 SUMMARY

Using COI sequences, we found high levels of species diversity for Collembola within the Namib Desert. The biogeographic data we provide demonstrated high levels of genetic variation and deeply divergent lineages that were restricted to local populations. We suggest that the high diversity of Namib Desert springtails is the result of long‐term isolation and accumulation of allopatric mutations in this extreme environment.

## AUTHOR CONTRIBUTIONS

G.E.C., I.D.H., and D.A.C. conceived of the project and experimental design. G.E.C., J.R.B., I.D.H., and D.A.C. conducted fieldwork. G.E.C. and J.R.B. processed samples for sequencing. G.E.C. performed all analyses and led the writing with assistance from I.D.H., D.A.C., and G. M.‐K.. G.E.C, J.R.B, I.D.H, D.A.C., and G. M‐K. provided input on the final version.

## Data Availability

Data available from the Dryad Digital Repository: https://doi.org/10.5061/dryad.2294qd9. DNA sequences: GenBank accessions to be added in proofs. Original trace files, assembled sequences (untrimmed), specimen images, collection information, and any other supporting information: Barcode of Life Datasystems (BOLD) online database (dataset DS‐NBCOL): DOI to be added in proofs. Full tables and the expanded phylogenetic tree are included as Supporting information.
