# Supplementary material for: Ancient landscapes of the Namib Desert harbor high levels of genetic variability and deeply divergent lineages for Collembola
Source: Ecol Evol. 2019 Apr 10;9(8):4969–79. doi: 10.1002/ece3.5103 (PMC6476783; doi:10.1002/ece3.5103)

Supporting Information

Appendix S1

Table S1.1: GPS details for sites A-R across the Gravel Plains of the Namib Desert from which springtails were found, successfully sequenced and presented in the current study. Where multiple soil samples within close proximity were merged, the mean GPS locality is provided in bold while specific locations of each soil sample (collection code) are subsequently provided below. Number of springtail sequences (*n*) and unique haplotypes (*h*) found from each site and sample are also listed. Collections containing the code ‘NB16‘ were taken in 2016, while ‘GINV’ and ‘GTRC’ samples were collected in 2015.

| **Site** | **Exact site** | **Collection code** | ***n*** | ***h*** | **Latitude** | **Longitude** | **Elevation (m)** |
| --- | --- | --- | --- | --- | --- | --- | --- |
| **A** | **C14-02** | **NB16-018** | **1** | **1** | **-23.00152** | **14.66968** | **121** |
| **B** | **C14-06** | **NB16-023** | **12** | **4** | **-23.06637** | **15.0421** | **539** |
| **C** |  |  | **27** | **14** | **-23.14309** | **15.20738** | **623** |
|  | C14-08 | NB16-025 | 7 | 4 | -23.14323 | 15.20747 | 624 |
|  |  | NB16-026 | 18 | 9 | -23.14322 | 15.20744 | 623 |
|  |  | NB16-027 | 2 | 1 | -23.14283 | 15.20722 | 623 |
| **D** |  |  | **15** | **9** | **-23.24377** | **15.35777** | **757** |
|  | C14-10 | NB16-028 | 11 | 7 | -23.24377 | 15.35777 | 757 |
|  |  | NB16-029 | 4 | 2 | -23.24377 | 15.35777 | 757 |
| **E** | **C14-12** | **NB16-033** | **3** | **3** | **-23.31041** | **15.53481** | **946** |
| **F** | **GINV12** |  | **1** | **2** | **-23.326** | **15.569** | **959** |
| **G** |  |  | **72** | **58** | **-23.32406** | **15.71384** | **897** |
|  | C14-14 | NB16-034 | 10 | 10 | -23.32463 | 15.71374 | 895 |
|  |  | NB16-035 | 11 | 9 | -23.32444 | 15.71357 | 898 |
|  |  | NB16-036 | 21 | 14 | -23.32319 | 15.7144 | 890 |
|  | GINV13 |  | 22 | 16 | -23.32359 | 15.71395 | 899 |
|  | GINV14 |  | 8 | 9 | -23.32445 | 15.71356 | 901 |
| **H** |  |  | **11** | **6** | **-23.32093** | **15.86175** | **969** |
|  | C14-16 | NB16-038 | 10 | 5 | -23.32093 | 15.86174 | 970 |
|  |  | NB16-039 | 1 | 1 | -23.32093 | 15.86176 | 967 |
| **I** | **C14-18** | **NB16-042** | **18** | **6** | **-23.34627** | **16.00951** | **1097** |
| **J** | **Site06** | **NB16-046** | **1** | **1** | **-23.30919** | **16.04211** | **1053** |
| **K** | **C14-20** |  | **49** | **23** | **-23.24533** | **16.14359** | **1268** |
|  |  | NB16-043 | 24 | 7 | -23.24535 | 16.14360 | 1266 |
|  |  | NB16-044 | 1 | 1 | -23.24533 | 16.14363 | 1269 |
|  |  | NB16-045 | 24 | 15 | -23.24530 | 16.14354 | 1268 |
| **L** | **Site07** | **NB16-047** | **24** | **18** | **-23.34888** | **15.55568** | **978** |
| **M** |  |  | **41** | **30** | **-23.391** | **15.529** | **884** |
|  | GINV02 |  | 6 | 4 | -23.391 | 15.529 | 879 |
|  | Site08 | NB16-048 | 35 | 26 | -23.39122 | 15.52974 | 888 |
| **N** | **GINV01** |  | **9** | **1** | **-23.456** | **15.378** | **774** |
| **O** | **Site02** |  | **18** | **7** | **-23.53272** | **15.18375** | **556** |
|  |  | NB16-005 | 12 | 4 | -23.53270 | 15.18375 | 556 |
|  |  | NB16-006 | 6 | 3 | -23.53273 | 15.18375 | 556 |
| **P** | **Site03** |  | **15** | **7** | **-23.54804** | **15.14017** | **518** |
|  |  | NB16-007 | 4 | 4 | -23.54805 | 15.14017 | 518 |
|  |  | NB16-008 | 11 | 3 | -23.54803 | 15.14017 | 518 |
| **Q** | **GTRC** | **GTRC** | **1** | **1** | **-23.56** | **15.038** | **406** |
| **R** | **Site13** | **NB16-055** | **23** | **4** | **-23.52144** | **15.04181** | **436** |
|  |  |  | **341** |  |  |  |  |

Appendix S2

Table S2.1: Collection details for each putative species of Namib Desert springtail, as identified by GMYC analyses, including the specific soil sample that specimens were isolated from (collection code), the site code used in the field (exact site) and the site letter used for analyses as presented in the current study (site). Barcode Identification Numbers (BINs) are also provided and are congruent with GMYC analyses in most cases. Asterisks indicate Non-isotomid taxa.

| GMYC species | BIN | Haplotype | Collection Code | Exact site | Site | n individuals |
| --- | --- | --- | --- | --- | --- | --- |
| 1 | ADB5701 | NC-038 | NB16-025 | C14-08 | C | 1 |
| 1 | ADB5701 | NC-053 | NB16-026 | C14-08 | C | 2 |
| 1 | ADB5701 | NC-054 | NB16-026 | C14-08 | C | 1 |
| 1 | ADB5701 | NC-055 | NB16-026 | C14-08 | C | 3 |
| 1 | ADB5701 | NC-056 | NB16-026 | C14-08 | C | 5 |
| 1 | ADB5701 | NC-058 | NB16-026 | C14-08 | C | 1 |
| 1 | ADB5701 | NC-162 | NB16-033 | C14-12 | E | 1 |
| 1 | ADB5701 | NC-084 | NB16-048 | Site08 | M | 1 |
| 1 | ADB5701 | NC-031 | NB16-006 | Site02 | O | 1 |
| 1 | ADB5701 | NC-032 | NB16-006 | Site02 | O | 1 |
| 1 | ADB5701 | NC-033 | NB16-005 | Site02 | O | 1 |
| 1 | ADB5701 | NC-033 | NB16-006 | Site02 | O | 4 |
| 1 | ADB5701 | NC-032 | NB16-007 | Site03 | P | 1 |
| 1 | ADB5701 | NC-074 | NB16-055 | Site13 | R | 15 |
| 1 | ADB5701 | NC-075 | NB16-055 | Site13 | R | 2 |
| 1 | ADB5701 | NC-088 | NB16-055 | Site13 | R | 4 |
| 1 | ADB5701 | NC-089 | NB16-055 | Site13 | R | 2 |
| 3 | ADB6305 | NC-039 | NB16-025 | C14-08 | C | 1 |
| 3 | ADB6305 | NC-039 | NB16-026 | C14-08 | C | 1 |
| 3 | ADB6305 | NC-040 | NB16-025 | C14-08 | C | 4 |
| 3 | ADB6305 | NC-040 | NB16-026 | C14-08 | C | 3 |
| 3 | ADB6305 | NC-040 | NB16-046 | Site06 | J | 1 |
| 3 | ADB6305 | NC-041 | NB16-025 | C14-08 | C | 1 |
| 2 | ADB6383 | NC-057 | NB16-026 | C14-08 | C | 1 |
| 2 | ADB6383 | NC-059 | NB16-026 | C14-08 | C | 1 |
| 2 | ADB6383 | NC-062 | NB16-047 | Site07 | L | 2 |
| 2 | ADB6383 | NC-164 | NB16-033 | C14-12 | E | 1 |
| 4 | ADB6302 | NC-099 | NB16-039 | C14-16 | H | 1 |
| 4 | ADB6302 | NC-101 | NB16-038 | C14-16 | H | 2 |
| 4 | ADB6302 | NC-152 | NB16-038 | C14-16 | H | 1 |
| 5 | ADB6299 | NC-100 | NB16-038 | C14-16 | H | 2 |
| 5 | ADB6301 | NC-102 | NB16-038 | C14-16 | H | 4 |
| 5 | ADB6555 | NC-151 | NB16-038 | C14-16 | H | 1 |
| 6 | ADC0536 | NC-129 | NB16-044 | C14-20 | K | 1 |
| 7 | ACU5054 | NC-012 | GINV14 | GINV14 | G | 1 |
| 7 | ACU5054 | NC-016 | GINV14 | GINV14 | G | 1 |
| 7 | ACU5054 | NC-109 | NB16-035 | C14-14 | G | 1 |
| 7 | ACU5054 | NC-110 | NB16-035 | C14-14 | G | 2 |
| 7 | ACU5054 | NC-111 | NB16-035 | C14-14 | G | 2 |
| 7 | ACU5054 | NC-112 | NB16-035 | C14-14 | G | 1 |
| 7 | ACU5054 | NC-113 | NB16-034 | C14-14 | G | 1 |
| 7 | ACU5054 | NC-113 | NB16-035 | C14-14 | G | 1 |
| 7 | ACU5054 | NC-114 | NB16-035 | C14-14 | G | 1 |
| 7 | ACU5054 | NC-115 | NB16-035 | C14-14 | G | 1 |
| 7 | ACU5054 | NC-116 | NB16-035 | C14-14 | G | 1 |
| 7 | ACU5054 | NC-130 | NB16-034 | C14-14 | G | 1 |
| 7 | ACU5054 | NC-137 | NB16-034 | C14-14 | G | 1 |
| 7 | ACU5054 | NC-153 | NB16-035 | C14-14 | G | 1 |
| 8 | ADB6378 | NC-093 | NB16-043 | C14-20 | K | 9 |
| 8 | ADB6378 | NC-093 | NB16-045 | C14-20 | K | 6 |
| 8 | ADB6300 | NC-094 | NB16-043 | C14-20 | K | 7 |
| 9 | ACU5273 | NC-013 | GINV14 | GINV14 | G | 1 |
| 9 | ACU5273 | NC-014 | GINV14 | GINV14 | G | 1 |
| 9 | ACU5273 | NC-015 | GINV14 | GINV14 | G | 1 |
| 9 | ACU5273 | NC-015 | NB16-005 | Site02 | O | 6 |
| 9 | ACU5273 | NC-017 | GINV14 | GINV14 | G | 1 |
| 9 | ACU5273 | NC-036 | NB16-005 | Site02 | O | 2 |
| 10 | ACU4213 | NC-006 | GINV12 | GINV12 | F | 1 |
| 10 | ACU4213 | NC-006 | GINV14 | GINV14 | G | 1 |
| 10 | ACU4213 | NC-018 | GINV14 | GINV14 | G | 1 |
| 10 | ACU4213 | NC-037 | NB16-005 | Site02 | O | 3 |
| 11 | ADC0533 | NC-118 | NB16-045 | C14-20 | K | 1 |
| 12 | ADB6633 | NC-047 | NB16-029 | C14-10 | D | 3 |
| 13 | ADC0535 | NC-120 | NB16-045 | C14-20 | K | 1 |
| 14 | ACU5084 | NC-001 | GINV01 | GINV01 | N | 9 |
| 14 | ACU5084 | NC-001 | GTRC | GTRC | Q | 1 |
| 14 | ACU5084 | NC-003 | GINV02 | GINV02 | M | 1 |
| 15 | ADB5714 | NC-095 | NB16-042 | C14-18 | I | 11 |
| 15 | ADB5714 | NC-096 | NB16-042 | C14-18 | I | 2 |
| 15 | ADB5714 | NC-149 | NB16-042 | C14-18 | I | 1 |
| 16 | ADB6379 | NC-119 | NB16-045 | C14-20 | K | 1 |
| 16 | ADB6379 | NC-138 | NB16-043 | C14-20 | K | 2 |
| 16 | ADB6379 | NC-138 | NB16-045 | C14-20 | K | 3 |
| 16 | ADB6379 | NC-140 | NB16-043 | C14-20 | K | 1 |
| 16 | ADB6379 | NC-140 | NB16-045 | C14-20 | K | 1 |
| 16 | ADB6379 | NC-141 | NB16-043 | C14-20 | K | 2 |
| 16 | ADB6379 | NC-141 | NB16-045 | C14-20 | K | 2 |
| 16 | ADB6379 | NC-143 | NB16-045 | C14-20 | K | 1 |
| 16 | ADB6379 | NC-144 | NB16-045 | C14-20 | K | 1 |
| 16 | ADB6379 | NC-145 | NB16-045 | C14-20 | K | 1 |
| 16 | ADB6379 | NC-146 | NB16-045 | C14-20 | K | 1 |
| 16 | ADB6379 | NC-147 | NB16-043 | C14-20 | K | 2 |
| 16 | ADB6379 | NC-148 | NB16-043 | C14-20 | K | 1 |
| 17 | ADC0534 | NC-117 | NB16-045 | C14-20 | K | 1 |
| 17 | ADC0534 | NC-121 | NB16-045 | C14-20 | K | 1 |
| 18 | ADB6047 | NC-103 | NB16-036 | C14-14 | G | 1 |
| 18 | ADB6047 | NC-104 | NB16-036 | C14-14 | G | 5 |
| 18 | ADB6047 | NC-105 | NB16-036 | C14-14 | G | 3 |
| 18 | ADB6047 | NC-106 | NB16-036 | C14-14 | G | 1 |
| 18 | ADB6047 | NC-107 | NB16-036 | C14-14 | G | 1 |
| 18 | ADB6047 | NC-108 | NB16-036 | C14-14 | G | 2 |
| 18 | ADB6047 | NC-154 | NB16-036 | C14-14 | G | 1 |
| 18 | ADB6047 | NC-155 | NB16-036 | C14-14 | G | 1 |
| 18 | ADB6047 | NC-156 | NB16-036 | C14-14 | G | 1 |
| 18 | ADB6047 | NC-157 | NB16-036 | C14-14 | G | 1 |
| 18 | ADB6047 | NC-158 | NB16-036 | C14-14 | G | 1 |
| 18 | ADB6047 | NC-159 | NB16-036 | C14-14 | G | 1 |
| 18 | ADB6047 | NC-160 | NB16-036 | C14-14 | G | 1 |
| 18 | ADB6047 | NC-161 | NB16-036 | C14-14 | G | 1 |
| 19 | ADB6381 | NC-034 | NB16-007 | Site03 | P | 1 |
| 20 | ADB6632 | NC-049 | NB16-023 | C14-06 | B | 8 |
| 20 | ADB6632 | NC-050 | NB16-023 | C14-06 | B | 1 |
| 20 | ADB6632 | NC-051 | NB16-023 | C14-06 | B | 2 |
| 20 | ADB6632 | NC-052 | NB16-023 | C14-06 | B | 1 |
| 20 | ADB6382 | NC-128 | NB16-027 | C14-08 | C | 2 |
| 20 | ADB6382 | NC-127 | NB16-028 | C14-10 | D | 1 |
| 20 | ADB6632 | NC-030 | NB16-007 | Site03 | P | 1 |
| 20 | ADB6632 | NC-030 | NB16-008 | Site03 | P | 9 |
| 20 | ADB6632 | NC-035 | NB16-007 | Site03 | P | 1 |
| 20 | ADB6632 | NC-086 | NB16-008 | Site03 | P | 1 |
| 20 | ADB6632 | NC-087 | NB16-008 | Site03 | P | 1 |
| 21 | ADB6303 | NC-142 | NB16-045 | C14-20 | K | 1 |
| 22 | ADB6384 | NC-046 | NB16-018 | C14-02 | A | 1 |
| 23 | ACU4253 | NC-004 | GINV02 | GINV02 | M | 2 |
| 24 | ADB6382 | NC-048 | NB16-028 | C14-10 | D | 3 |
| 24 | ADB6382 | NC-048 | NB16-029 | C14-10 | D | 1 |
| 24 | ADB6382 | NC-122 | NB16-028 | C14-10 | D | 2 |
| 24 | ADB6382 | NC-123 | NB16-028 | C14-10 | D | 2 |
| 24 | ADB6382 | NC-124 | NB16-028 | C14-10 | D | 1 |
| 24 | ADB6382 | NC-125 | NB16-028 | C14-10 | D | 1 |
| 24 | ADB6382 | NC-126 | NB16-028 | C14-10 | D | 1 |
| 25 | ACU4405 | NC-005 | GINV02 | GINV02 | M | 1 |
| 25 | ACU4405 | NC-042 | NB16-048 | Site08 | M | 2 |
| 25 | ACU4405 | NC-042 | NB16-047 | Site07 | L | 1 |
| 25 | ACU4405 | NC-043 | NB16-048 | Site08 | M | 2 |
| 25 | ACU4405 | NC-060 | NB16-047 | Site07 | L | 1 |
| 25 | ACU4405 | NC-061 | NB16-047 | Site07 | L | 3 |
| 25 | ACU4405 | NC-063 | NB16-047 | Site07 | L | 1 |
| 25 | ACU4405 | NC-064 | NB16-047 | Site07 | L | 2 |
| 25 | ACU4405 | NC-073 | NB16-047 | Site07 | L | 1 |
| 25 | ACU4405 | NC-076 | NB16-048 | Site08 | M | 1 |
| 25 | ACU4405 | NC-077 | NB16-048 | Site08 | M | 2 |
| 25 | ACU4405 | NC-078 | NB16-048 | Site08 | M | 3 |
| 25 | ACU4405 | NC-080 | NB16-048 | Site08 | M | 1 |
| 25 | ACU4405 | NC-081 | NB16-048 | Site08 | M | 1 |
| 25 | ACU4405 | NC-082 | NB16-048 | Site08 | M | 1 |
| 25 | ACU4405 | NC-085 | NB16-048 | Site08 | M | 1 |
| 25 | ACU4405 | NC-163 | NB16-033 | C14-12 | E | 1 |
| 25 | ACU4405 | NC-165 | NB16-048 | Site08 | M | 1 |
| 25 | ACU4405 | NC-168 | NB16-048 | Site08 | M | 1 |
| 25 | ACU4405 | NC-169 | NB16-048 | Site08 | M | 1 |
| 25 | ACU4405 | NC-171 | NB16-048 | Site08 | M | 1 |
| 25 | ACU4405 | NC-173 | NB16-048 | Site08 | M | 1 |
| 25 | ACU4405 | NC-174 | NB16-048 | Site08 | M | 1 |
| 25 | ACU4405 | NC-175 | NB16-048 | Site08 | M | 1 |
| 26 | ACU4502 | NC-065 | NB16-047 | Site07 | L | 2 |
| 26 | ACU4502 | NC-066 | NB16-047 | Site07 | L | 1 |
| 26 | ACU4502 | NC-067 | NB16-047 | Site07 | L | 1 |
| 26 | ACU4502 | NC-068 | NB16-047 | Site07 | L | 1 |
| 26 | ACU4502 | NC-069 | NB16-047 | Site07 | L | 1 |
| 26 | ACU4502 | NC-070 | NB16-047 | Site07 | L | 1 |
| 26 | ACU4502 | NC-071 | NB16-047 | Site07 | L | 1 |
| 26 | ACU4502 | NC-072 | NB16-047 | Site07 | L | 1 |
| 26 | ACU4502 | NC-090 | NB16-047 | Site07 | L | 2 |
| 26 | ACU4502 | NC-091 | NB16-047 | Site07 | L | 1 |
| 26 | ACU4502 | NC-092 | NB16-047 | Site07 | L | 1 |
| 26 | ACU4502 | NC-002 | GINV02 | GINV02 | M | 2 |
| 26 | ACU4502 | NC-002 | NB16-048 | Site08 | M | 1 |
| 26 | ACU4502 | NC-044 | NB16-048 | Site08 | M | 1 |
| 26 | ACU4502 | NC-045 | NB16-048 | Site08 | M | 1 |
| 26 | ACU4502 | NC-079 | NB16-048 | Site08 | M | 1 |
| 26 | ACU4502 | NC-083 | NB16-048 | Site08 | M | 4 |
| 26 | ACU4502 | NC-166 | NB16-048 | Site08 | M | 2 |
| 26 | ACU4502 | NC-167 | NB16-048 | Site08 | M | 1 |
| 26 | ACU4502 | NC-170 | NB16-048 | Site08 | M | 1 |
| 26 | ACU4502 | NC-172 | NB16-048 | Site08 | M | 1 |
| 26 | ACU5164 | NC-007 | GINV13 | GINV13 | G | 1 |
| 26 | ACU5164 | NC-020 | GINV13 | GINV13 | G | 1 |
| 26 | ACU5164 | NC-026 | GINV13 | GINV13 | G | 1 |
| 26 | ACU5165 | NC-009 | GINV13 | GINV13 | G | 1 |
| 26 | ACU5165 | NC-027 | GINV13 | GINV13 | G | 1 |
| 26 | ACU5166 | NC-008 | GINV13 | GINV13 | G | 1 |
| 26 | ACU5166 | NC-011 | GINV13 | GINV13 | G | 1 |
| 26 | ACU5166 | NC-028 | GINV13 | GINV13 | G | 1 |
| 26 | ACW5240 | NC-019 | GINV13 | GINV13 | G | 1 |
| 26 | ACW5553 | NC-029 | GINV13 | GINV13 | G | 1 |
| 26 | ACW5553 | NC-133 | NB16-034 | C14-14 | G | 1 |
| 26 | ACW5554 | NC-025 | GINV13 | GINV13 | G | 1 |
| 26 | ACW5606 | NC-024 | GINV13 | GINV13 | G | 3 |
| 26 | ACW5607 | NC-023 | NB16-034 | C14-14 | G | 1 |
| 26 | ACW5607 | NC-023 | GINV13 | GINV13 | G | 2 |
| 26 | ACW5608 | NC-010 | GINV13 | GINV13 | G | 2 |
| 26 | ACW5608 | NC-022 | GINV13 | GINV13 | G | 3 |
| 26 | ACW5608 | NC-131 | NB16-034 | C14-14 | G | 1 |
| 26 | ACW5608 | NC-132 | NB16-034 | C14-14 | G | 1 |
| 26 | ACW5608 | NC-134 | NB16-034 | C14-14 | G | 1 |
| 26 | ACW5608 | NC-136 | NB16-034 | C14-14 | G | 1 |
| 26 | ADB9904 | NC-135 | NB16-034 | C14-14 | G | 1 |
| 27* | ACW5615 | NC-021 | GINV13 | GINV13 | G | 1 |
| 28* | ADB6773 | NC-150 | NB16-042 | C14-18 | I | 1 |
| 29* | ADC0483 | NC-097 | NB16-042 | C14-18 | I | 2 |
| 29* | ADC0483 | NC-098 | NB16-042 | C14-18 | I | 1 |
| 30* | ADB6048 | NC-139 | NB16-045 | C14-20 | K | 2 |

Table S2.2 Collection details for each Namib Desert springtail COI haplotype, including the specific soil sample that specimens were isolated from (collection code), the site code used in the field (exact site) and the site letter used for analyses as presented in the current study (sites). Barcode Identification Numbers (BINs) are also provided.

| **Haplotype** | **n sequences** | **BIN** | **Sites** | **n sequences** | **Collection Code** | **Exact Site** |
| --- | --- | --- | --- | --- | --- | --- |
| NC-001 | 10 | BOLD:ACU5084 | N | 9 | GINV01 | GINV01 |
|  |  |  | Q | 1 | GTRC | GTRC |
| NC-002 | 3 | BOLD:ACU4502 | M | 2 | GINV02 | GINV02 |
|  |  |  |  | 1 | NB16-048 | Site08 |
| NC-003 | 1 | BOLD:ACU5084 | M | 1 | GINV02 | GINV02 |
| NC-004 | 2 | BOLD:ACU4253 | M | 2 | GINV02 | GINV02 |
| NC-005 | 1 | BOLD:ACU4405 | M | 1 | GINV02 | GINV02 |
| NC-006 | 2 | BOLD:ACU4213 | F | 1 | GINV12 | GINV12 |
|  |  |  | G | 1 | GINV14 | GINV14 |
| NC-007 | 1 | BOLD:ACU5164 | G | 1 | GINV13 | GINV13 |
| NC-008 | 1 | BOLD:ACU5166 | G | 1 | GINV13 | GINV13 |
| NC-009 | 1 | BOLD:ACU5165 | G | 1 | GINV13 | GINV13 |
| NC-010 | 2 | BOLD:ACW5608 | G | 2 | GINV13 | GINV13 |
| NC-011 | 1 | BOLD:ACU5166 | G | 1 | GINV13 | GINV13 |
| NC-012 | 1 | BOLD:ACU5054 | G | 1 | GINV14 | GINV14 |
| NC-013 | 1 | BOLD:ACU5273 | G | 1 | GINV14 | GINV14 |
| NC-014 | 1 | BOLD:ACU5273 | G | 1 | GINV14 | GINV14 |
| NC-015 | 7 | BOLD:ACU5273 | G | 1 | GINV14 | GINV14 |
|  |  |  | O | 6 | NB16-005 | Site02 |
| NC-016 | 1 | BOLD:ACU5054 | G | 1 | GINV14 | GINV14 |
| NC-017 | 1 | BOLD:ACU5273 | G | 1 | GINV14 | GINV14 |
| NC-018 | 1 | BOLD:ACU4213 | G | 1 | GINV14 | GINV14 |
| NC-019 | 1 | BOLD:ACW5240 | G | 1 | GINV13 | GINV13 |
| NC-020 | 1 | BOLD:ACU5164 | G | 1 | GINV13 | GINV13 |
| NC-021 | 1 | BOLD:ACW5615 | G | 1 | GINV13 | GINV13 |
| NC-022 | 3 | BOLD:ACW5608 | G | 3 | GINV13 | GINV13 |
| NC-023 | 3 | BOLD:ACW5607 | G | 1 | NB16-034 | C14-14 |
|  |  |  |  | 2 | GINV13 | GINV13 |
| NC-024 | 3 | BOLD:ACW5606 | G | 3 | GINV13 | GINV13 |
| NC-025 | 1 | BOLD:ACW5554 | G | 1 | GINV13 | GINV13 |
| NC-026 | 1 | BOLD:ACU5164 | G | 1 | GINV13 | GINV13 |
| NC-027 | 1 | BOLD:ACU5165 | G | 1 | GINV13 | GINV13 |
| NC-028 | 1 | BOLD:ACU5166 | G | 1 | GINV13 | GINV13 |
| NC-029 | 1 | BOLD:ACW5553 | G | 1 | GINV13 | GINV13 |
| NC-030 | 10 | BOLD:ADB6632 | P | 1 | NB16-007 | Site03 |
|  |  |  |  | 9 | NB16-008 | Site03 |
| NC-031 | 1 | BOLD:ADB5701 | O | 1 | NB16-006 | Site02 |
| NC-032 | 2 | BOLD:ADB5701 | O | 1 | NB16-006 | Site02 |
|  |  |  | P | 1 | NB16-007 | Site03 |
| NC-033 | 5 | BOLD:ADB5701 | O | 1 | NB16-005 | Site02 |
|  |  |  |  | 4 | NB16-006 | Site02 |
| NC-034 | 1 | BOLD:ADB6381 | P | 1 | NB16-007 | Site03 |
| NC-035 | 1 | BOLD:ADB6632 | P | 1 | NB16-007 | Site03 |
| NC-036 | 2 | BOLD:ACU5273 | O | 2 | NB16-005 | Site02 |
| NC-037 | 3 | BOLD:ACU4213 | O | 3 | NB16-005 | Site02 |
| NC-038 | 1 | BOLD:ADB5701 | C | 1 | NB16-025 | C14-08 |
| NC-039 | 2 | BOLD:ADB6305 | C | 1 | NB16-025 | C14-08 |
|  |  |  | C | 1 | NB16-026 | C14-08 |
| NC-040 | 8 | BOLD:ADB6305 | C | 4 | NB16-025 | C14-08 |
|  |  |  |  | 3 | NB16-026 | C14-08 |
|  |  |  | J | 1 | NB16-046 | Site06 |
| NC-041 | 1 | BOLD:ADB6305 | C | 1 | NB16-025 | C14-08 |
| NC-042 | 3 | BOLD:ACU4405 | L | 1 | NB16-047 | Site07 |
|  |  |  | M | 2 | NB16-048 | Site08 |
| NC-043 | 2 | BOLD:ACU4405 | M | 2 | NB16-048 | Site08 |
| NC-044 | 1 | BOLD:ACU4502 | M | 1 | NB16-048 | Site08 |
| NC-045 | 1 | BOLD:ACU4502 | M | 1 | NB16-048 | Site08 |
| NC-046 | 1 | BOLD:ADB6384 | A | 1 | NB16-018 | C14-02 |
| NC-047 | 3 | BOLD:ADB6633 | D | 3 | NB16-029 | C14-10 |
| NC-048 | 4 | BOLD:ADB6382 | D | 3 | NB16-028 | C14-10 |
|  |  |  |  | 1 | NB16-029 | C14-10 |
| NC-049 | 8 | BOLD:ADB6632 | B | 8 | NB16-023 | C14-06 |
| NC-050 | 1 | BOLD:ADB6632 | B | 1 | NB16-023 | C14-06 |
| NC-051 | 2 | BOLD:ADB6632 | B | 2 | NB16-023 | C14-06 |
| NC-052 | 1 | BOLD:ADB6632 | B | 1 | NB16-023 | C14-06 |
| NC-053 | 2 | BOLD:ADB5701 | C | 2 | NB16-026 | C14-08 |
| NC-054 | 1 | BOLD:ADB5701 | C | 1 | NB16-026 | C14-08 |
| NC-055 | 3 | BOLD:ADB5701 | C | 3 | NB16-026 | C14-08 |
| NC-056 | 5 | BOLD:ADB5701 | C | 5 | NB16-026 | C14-08 |
| NC-057 | 1 | BOLD:ADB6383 | C | 1 | NB16-026 | C14-08 |
| NC-058 | 1 | BOLD:ADB5701 | C | 1 | NB16-026 | C14-08 |
| NC-059 | 1 | BOLD:ADB6383 | C | 1 | NB16-026 | C14-08 |
| NC-060 | 1 | BOLD:ACU4405 | L | 1 | NB16-047 | Site07 |
| NC-061 | 3 | BOLD:ACU4405 | L | 3 | NB16-047 | Site07 |
| NC-062 | 2 | BOLD:ADB6383 | L | 2 | NB16-047 | Site07 |
| NC-063 | 1 | BOLD:ACU4405 | L | 1 | NB16-047 | Site07 |
| NC-064 | 2 | BOLD:ACU4405 | L | 2 | NB16-047 | Site07 |
| NC-065 | 2 | BOLD:ACU4502 | L | 2 | NB16-047 | Site07 |
| NC-066 | 1 | BOLD:ACU4502 | L | 1 | NB16-047 | Site07 |
| NC-067 | 1 | BOLD:ACU4502 | L | 1 | NB16-047 | Site07 |
| NC-068 | 1 | BOLD:ACU4502 | L | 1 | NB16-047 | Site07 |
| NC-069 | 1 | BOLD:ACU4502 | L | 1 | NB16-047 | Site07 |
| NC-070 | 1 | BOLD:ACU4502 | L | 1 | NB16-047 | Site07 |
| NC-071 | 1 | BOLD:ACU4502 | L | 1 | NB16-047 | Site07 |
| NC-072 | 1 | BOLD:ACU4502 | L | 1 | NB16-047 | Site07 |
| NC-073 | 1 | BOLD:ACU4405 | L | 1 | NB16-047 | Site07 |
| NC-074 | 15 | BOLD:ADB5701 | R | 15 | NB16-055 | Site13 |
| NC-075 | 2 | BOLD:ADB5701 | R | 2 | NB16-055 | Site13 |
| NC-076 | 1 | BOLD:ACU4405 | M | 1 | NB16-048 | Site08 |
| NC-077 | 2 | BOLD:ACU4405 | M | 2 | NB16-048 | Site08 |
| NC-078 | 3 | BOLD:ACU4405 | M | 3 | NB16-048 | Site08 |
| NC-079 | 1 | BOLD:ACU4502 | M | 1 | NB16-048 | Site08 |
| NC-080 | 1 | BOLD:ACU4405 | M | 1 | NB16-048 | Site08 |
| NC-081 | 1 | BOLD:ACU4405 | M | 1 | NB16-048 | Site08 |
| NC-082 | 1 | BOLD:ACU4405 | M | 1 | NB16-048 | Site08 |
| NC-083 | 4 | BOLD:ACU4502 | M | 4 | NB16-048 | Site08 |
| NC-084 | 1 | BOLD:ADB5701 | M | 1 | NB16-048 | Site08 |
| NC-085 | 1 | BOLD:ACU4405 | M | 1 | NB16-048 | Site08 |
| NC-086 | 1 | BOLD:ADB6632 | P | 1 | NB16-008 | Site03 |
| NC-087 | 1 | BOLD:ADB6632 | P | 1 | NB16-008 | Site03 |
| NC-088 | 4 | BOLD:ADB5701 | R | 4 | NB16-055 | Site13 |
| NC-089 | 2 | BOLD:ADB5701 | R | 2 | NB16-055 | Site13 |
| NC-090 | 2 | BOLD:ACU4502 | L | 2 | NB16-047 | Site07 |
| NC-091 | 1 | BOLD:ACU4502 | L | 1 | NB16-047 | Site07 |
| NC-092 | 1 | BOLD:ACU4502 | L | 1 | NB16-047 | Site07 |
| NC-093 | 15 | BOLD:ADB6378 | K | 9 | NB16-043 | C14-20 |
|  |  |  |  | 6 | NB16-045 | C14-20 |
| NC-094 | 7 | BOLD:ADB6300 | K | 7 | NB16-043 | C14-20 |
| NC-095 | 11 | BOLD:ADB5714 | I | 11 | NB16-042 | C14-18 |
| NC-096 | 2 | BOLD:ADB5714 | I | 2 | NB16-042 | C14-18 |
| NC-097 | 2 | BOLD:ADC0483 | I | 2 | NB16-042 | C14-18 |
| NC-098 | 1 | BOLD:ADC0483 | I | 1 | NB16-042 | C14-18 |
| NC-099 | 1 | BOLD:ADB6302 | H | 1 | NB16-039 | C14-16 |
| NC-100 | 2 | BOLD:ADB6299 | H | 2 | NB16-038 | C14-16 |
| NC-101 | 2 | BOLD:ADB6302 | H | 2 | NB16-038 | C14-16 |
| NC-102 | 4 | BOLD:ADB6301 | H | 4 | NB16-038 | C14-16 |
| NC-103 | 1 | BOLD:ADB6047 | G | 1 | NB16-036 | C14-14 |
| NC-104 | 5 | BOLD:ADB6047 | G | 5 | NB16-036 | C14-14 |
| NC-105 | 3 | BOLD:ADB6047 | G | 3 | NB16-036 | C14-14 |
| NC-106 | 1 | BOLD:ADB6047 | G | 1 | NB16-036 | C14-14 |
| NC-107 | 1 | BOLD:ADB6047 | G | 1 | NB16-036 | C14-14 |
| NC-108 | 2 | BOLD:ADB6047 | G | 2 | NB16-036 | C14-14 |
| NC-109 | 1 | BOLD:ACU5054 | G | 1 | NB16-035 | C14-14 |
| NC-110 | 2 | BOLD:ACU5054 | G | 2 | NB16-035 | C14-14 |
| NC-111 | 2 | BOLD:ACU5054 | G | 2 | NB16-035 | C14-14 |
| NC-112 | 1 | BOLD:ACU5054 | G | 1 | NB16-035 | C14-14 |
| NC-113 | 2 | BOLD:ACU5054 | G | 1 | NB16-034 | C14-14 |
|  |  |  |  | 1 | NB16-035 | C14-14 |
| NC-114 | 1 | BOLD:ACU5054 | G | 1 | NB16-035 | C14-14 |
| NC-115 | 1 | BOLD:ACU5054 | G | 1 | NB16-035 | C14-14 |
| NC-116 | 1 | BOLD:ACU5054 | G | 1 | NB16-035 | C14-14 |
| NC-117 | 1 | BOLD:ADC0534 | K | 1 | NB16-045 | C14-20 |
| NC-118 | 1 | BOLD:ADC0533 | K | 1 | NB16-045 | C14-20 |
| NC-119 | 1 | BOLD:ADB6379 | K | 1 | NB16-045 | C14-20 |
| NC-120 | 1 | BOLD:ADC0535 | K | 1 | NB16-045 | C14-20 |
| NC-121 | 1 | BOLD:ADC0534 | K | 1 | NB16-045 | C14-20 |
| NC-122 | 2 | BOLD:ADB6382 | D | 2 | NB16-028 | C14-10 |
| NC-123 | 2 | BOLD:ADB6382 | D | 2 | NB16-028 | C14-10 |
| NC-124 | 1 | BOLD:ADB6382 | D | 1 | NB16-028 | C14-10 |
| NC-125 | 1 | BOLD:ADB6382 | D | 1 | NB16-028 | C14-10 |
| NC-126 | 1 | BOLD:ADB6382 | D | 1 | NB16-028 | C14-10 |
| NC-127 | 1 | BOLD:ADB6382 | D | 1 | NB16-028 | C14-10 |
| NC-128 | 2 | BOLD:ADB6382 | C | 2 | NB16-027 | C14-08 |
| NC-129 | 1 | BOLD:ADC0536 | K | 1 | NB16-044 | C14-20 |
| NC-130 | 1 | BOLD:ACU5054 | G | 1 | NB16-034 | C14-14 |
| NC-131 | 1 | BOLD:ACW5608 | G | 1 | NB16-034 | C14-14 |
| NC-132 | 1 | BOLD:ACW5608 | G | 1 | NB16-034 | C14-14 |
| NC-133 | 1 | BOLD:ACW5553 | G | 1 | NB16-034 | C14-14 |
| NC-134 | 1 | BOLD:ACW5608 | G | 1 | NB16-034 | C14-14 |
| NC-135 | 1 | BOLD:ADB9904 | G | 1 | NB16-034 | C14-14 |
| NC-136 | 1 | BOLD:ACW5608 | G | 1 | NB16-034 | C14-14 |
| NC-137 | 1 | BOLD:ACU5054 | G | 1 | NB16-034 | C14-14 |
| NC-138 | 5 | BOLD:ADB6379 | K | 2 | NB16-043 | C14-20 |
|  |  |  |  | 3 | NB16-045 | C14-20 |
| NC-139 | 2 | BOLD:ADB6048 | K | 2 | NB16-045 | C14-20 |
| NC-140 | 2 | BOLD:ADB6379 | K | 1 | NB16-043 | C14-20 |
|  |  |  |  | 1 | NB16-045 | C14-20 |
| NC-141 | 4 | BOLD:ADB6379 | K | 2 | NB16-043 | C14-20 |
|  |  |  |  | 2 | NB16-045 | C14-20 |
| NC-142 | 1 | BOLD:ADB6303 | K | 1 | NB16-045 | C14-20 |
| NC-143 | 1 | BOLD:ADB6379 | K | 1 | NB16-045 | C14-20 |
| NC-144 | 1 | BOLD:ADB6379 | K | 1 | NB16-045 | C14-20 |
| NC-145 | 1 | BOLD:ADB6379 | K | 1 | NB16-045 | C14-20 |
| NC-146 | 1 | BOLD:ADB6379 | K | 1 | NB16-045 | C14-20 |
| NC-147 | 2 | BOLD:ADB6379 | K | 2 | NB16-043 | C14-20 |
| NC-148 | 1 | BOLD:ADB6379 | K | 1 | NB16-043 | C14-20 |
| NC-149 | 1 | BOLD:ADB5714 | I | 1 | NB16-042 | C14-18 |
| NC-150 | 1 | BOLD:ADB6773 | I | 1 | NB16-042 | C14-18 |
| NC-151 | 1 | BOLD:ADB6555 | H | 1 | NB16-038 | C14-16 |
| NC-152 | 1 | BOLD:ADB6302 | H | 1 | NB16-038 | C14-16 |
| NC-153 | 1 | BOLD:ACU5054 | G | 1 | NB16-035 | C14-14 |
| NC-154 | 1 | BOLD:ADB6047 | G | 1 | NB16-036 | C14-14 |
| NC-155 | 1 | BOLD:ADB6047 | G | 1 | NB16-036 | C14-14 |
| NC-156 | 1 | BOLD:ADB6047 | G | 1 | NB16-036 | C14-14 |
| NC-157 | 1 | BOLD:ADB6047 | G | 1 | NB16-036 | C14-14 |
| NC-158 | 1 | BOLD:ADB6047 | G | 1 | NB16-036 | C14-14 |
| NC-159 | 1 | BOLD:ADB6047 | G | 1 | NB16-036 | C14-14 |
| NC-160 | 1 | BOLD:ADB6047 | G | 1 | NB16-036 | C14-14 |
| NC-161 | 1 | BOLD:ADB6047 | G | 1 | NB16-036 | C14-14 |
| NC-162 | 1 | BOLD:ADB5701 | E | 1 | NB16-033 | C14-12 |
| NC-163 | 1 | BOLD:ACU4405 | E | 1 | NB16-033 | C14-12 |
| NC-164 | 1 | BOLD:ADB6383 | E | 1 | NB16-033 | C14-12 |
| NC-165 | 1 | BOLD:ACU4405 | M | 1 | NB16-048 | Site08 |
| NC-166 | 2 | BOLD:ACU4502 | M | 2 | NB16-048 | Site08 |
| NC-167 | 1 | BOLD:ACU4502 | M | 1 | NB16-048 | Site08 |
| NC-168 | 1 | BOLD:ACU4405 | M | 1 | NB16-048 | Site08 |
| NC-169 | 1 | BOLD:ACU4405 | M | 1 | NB16-048 | Site08 |
| NC-170 | 1 | BOLD:ACU4502 | M | 1 | NB16-048 | Site08 |
| NC-171 | 1 | BOLD:ACU4405 | M | 1 | NB16-048 | Site08 |
| NC-172 | 1 | BOLD:ACU4502 | M | 1 | NB16-048 | Site08 |
| NC-173 | 1 | BOLD:ACU4405 | M | 1 | NB16-048 | Site08 |
| NC-174 | 1 | BOLD:ACU4405 | M | 1 | NB16-048 | Site08 |
| NC-175 | 1 | BOLD:ACU4405 | M | 1 | NB16-048 | Site08 |

Appendix S3

Table S3.1: List of Namib Desert soil samples from which no springtails (Collembola) were found.

| Collection code | Exact site | Date of sampling | Latitude | Longitude |
| --- | --- | --- | --- | --- |
| NB16-001 | site 1 | 11/04/2016 | -23.53026 | 15.18775 |
| NB16-002 | site 1 | 11/04/2016 | -23.53049 | 15.18777 |
| NB16-003 | site 1 | 11/04/2016 | -23.5305 | 15.18779 |
| NB16-004 | site 1 | 11/04/2016 | -23.53008 | 15.1881 |
| NB16-009 | site 4 | 11/04/2016 | -23.55011 | 15.12525 |
| NB16-010 | site 4 | 11/04/2016 | -23.55022 | 15.12536 |
| NB16-011 | site 4 | 11/04/2016 | -23.55021 | 15.12541 |
| NB16-012 | site 4 | 11/04/2016 | -23.5502 | 15.1254 |
| NB16-013 | site 5 | 11/04/2016 | -23.601611 | 15.013663 |
| NB16-014 | site 5 | 11/04/2016 | -23.60341 | 15.012471 |
| NB16-015 | site 5 | 11/04/2016 | -23.599984 | 15.015102 |
| NB16-016 | C14-2 | 12/04/2016 | -23.00179 | 14.66979 |
| NB16-017 | C14-2 | 12/04/2016 | -23.00201 | 14.66968 |
| NB16-020 | C14-4 | 12/04/2016 | -23.01642 | 14.86028 |
| NB16-021 | C14-4 | 12/04/2016 | -23.0166 | 14.86043 |
| NB16-024 | C14-6 | 12/04/2016 | -23.06649 | 15.04105 |
| NB16-030 | C14-10 | 12/04/2016 | -23.24384 | 15.35814 |
| NB16-031 | C14-12 | 12/04/2016 | -23.31069 | 15.53458 |
| NB16-032 | C14-12 | 12/04/2016 | -23.3106 | 15.5347 |
| NB16-040 | C14-18 | 12/04/2016 | -23.34629 | 16.00949 |
| NB16-041 | C14-18 | 12/04/2016 | -23.34627 | 16.00951 |
| NB16-049 | site 9 | 14/04/2016 | -23.05912 | 14.63217 |
| NB16-050 | C14-2 | 14/04/2016 | -23.00158 | 14.66973 |
| NB16-051 | C14-4 | 14/04/2016 | -23.01869 | 14.86174 |
| NB16-053 | site 11 | 14/04/2016 | -23.32953 | 15.04061 |
| NB16-056 | site 14 | 15/04/2016 | -22.34256 | 14.43123 |
| NB16-057 | site 15 | 15/04/2016 | -21.59415 | 13.89245 |
| NB16-058 | site 16 | 15/04/2016 | -21.19316 | 13.67546 |
| NB16-059 | site 17 | 15/04/2016 | -21.74357 | 13.99374 |

Table S3.2: List of Namib Desert soil samples where springtails were found but failed to sequence.

| Collection code | Exact site | Number of springtails extracted (failed to sequence) | Latitude | Longitude |
| --- | --- | --- | --- | --- |
| NB16-022 | C14-6 | 12 | -23.06619 | 15.04038 |
| NB16-019 | C14-4 | 4 | -23.0169 | 14.86088 |
| NB16-054 | site 12 | 3 | -23.4354 | 15.04116 |
| NB16-052 | site 10 | 2 | -23.12199 | 15.03172 |
| NB16-037 | C14-16 | 1 | -23.32093 | 15.86166 |

Appendix S4

Figure S4.1: Full Maximum Likelihood phylogenetic tree (GTR+I+G; 582 bp) as created in MEGA v7 showing grouping of unique haplotypes (n = 175) into their respective putative species-level designations, as determined by GMYC analyses, with bootstrap support (1000 replicates) at nodes. .


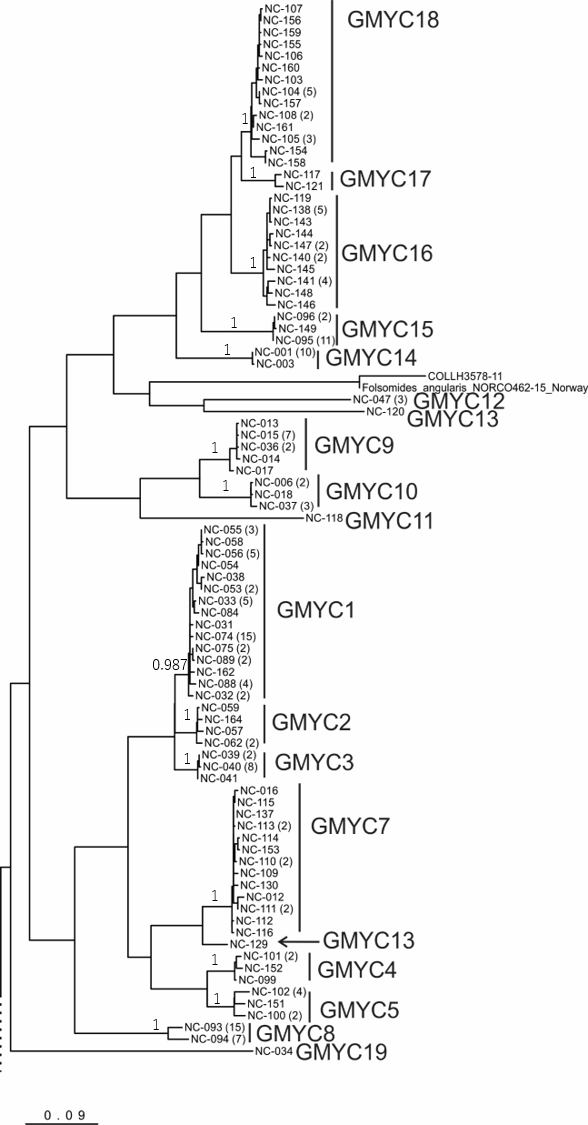

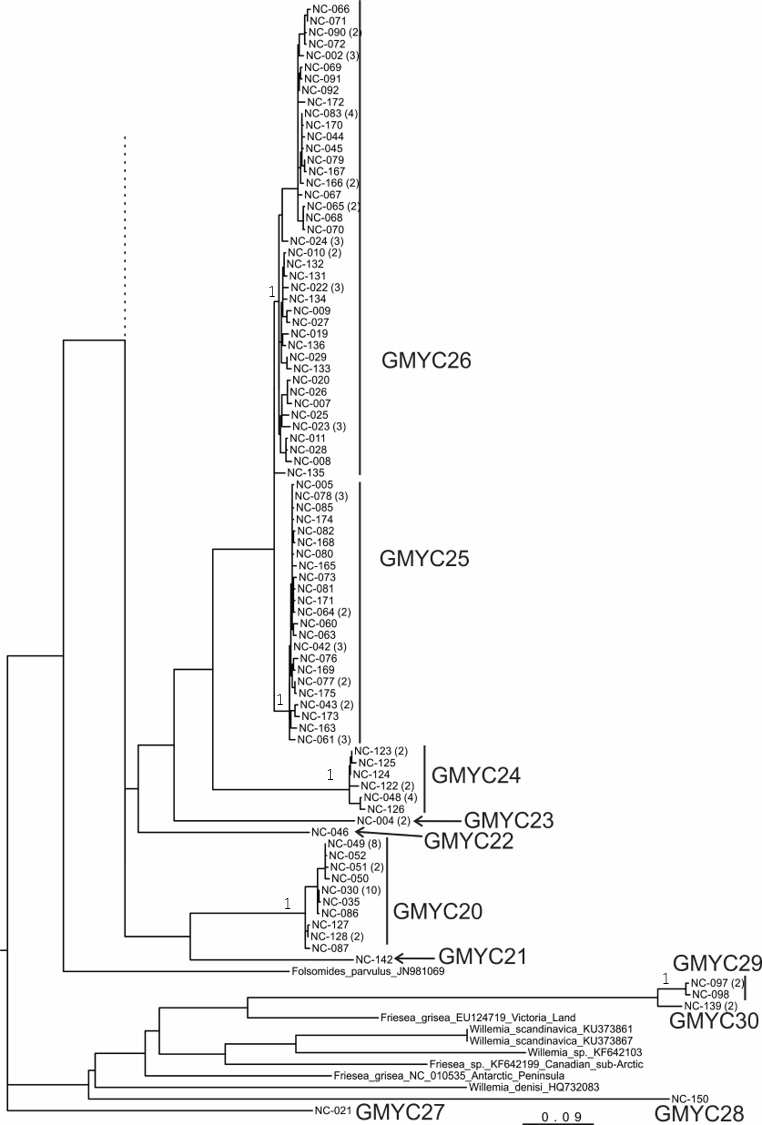

Supplement: Supplementary file 1 [file ECE3-9-4969-s001.docx]
